# Characterization of H5N1 influenza viruses isolated from humans *in vitro*

**DOI:** 10.1186/1743-422X-7-112

**Published:** 2010-06-01

**Authors:** Yong-Gang Li, Malinee Chittaganpitch, Sunthareeya Waicharoen, Yuta Kanai, Gui-Rong Bai, Masanori Kameoka, Naokazu Takeda, Kazuyoshi Ikuta, Pathom Sawanpanyalert

**Affiliations:** 1 Section of Viral Infections, Thailand-Japan Research Collaboration Center on Emerging and Re-emerging Infections, Tiwanon Road, Muang, Nonthaburi 11000, Thailand; 2 Department of Virology, Research Institute for Microbial Diseases, Osaka University, Yamadaoka 3-1, Suita, Osaka 565-0871, Japan; 3 Department of Influenza Virus, National Institute of Health, Department of Medical Sciences, Ministry of Public Health, Tiwanon Road, Muang, Nonthaburi 11000, Thailand

## Abstract

Since December 1997, highly pathogenic avian influenza A H5N1viruses have swept through poultry populations across Asian countries and been transmitted into African and European countries. We characterized 6 avian influenza H5N1 viruses isolated from humans in 2004 in Thailand. A highly pathogenic (HP) KAN353 strain showed faster replication and higher virulence in embryonated eggs compared to other strains, especially compared to the low pathogenic (LP) SP83 strain. HP KAN353 also showed strong cytopathogenicity compared to SP83 in Madin-Darby canine kidney cells. Interestingly, LP SP83 induced smaller plaques compared to other strains, especially HP KAN353. PB2 amino acid 627E may contribute to low virulence, whereas either PB2 amino acid 627 K or the combination of 627E/701N seems to be associated with high virulence. The *in vitro *assays used in this study may provide the basis for assessing the pathogenesis of influenza H5N1 viruses *in vivo*.

## Introduction

H5N1 avian influenza viruses are a causative agent of outbreaks of fatal disease in poultry worldwide, and a cause of fatal infection in humans with a more than 50% mortality rate since 1997 [1,2]http://www.who.int/csr/disease/avian_influenza/country/cases_table_2009_08_11/en/index.htm. Despite culling of all poultry on farms and probable eradication of the index genotype, novel genotypes have emerged [3]. Since 2004, the Z genotype has become dominant and spread to Southeast Asian countries including Thailand, Vietnam, Cambodia, and Laos [1]. Recently, genotype Z H5N1 viruses have been detected in domestic and wild birds in Central Asia, the Middle East, Africa and Europe, and migratory waterfowl have been implicated in the geographic expansion of the disease [4]. As of August 2009, the cumulative number of confirmed human cases of avian H5N1 influenza reported to the WHO was 438, 262 of which died http://www.who.int/csr/disease/avian_influenza/country/cases_table_2009_08_11/en/index.htm. It is important to elucidate the genetic determinants that allow cross species transfer of avian influenza viruses into mammalian populations and to elucidate the molecular basis of the pathogenicity in mammals, since H5N1 viruses isolated from humans in 1997 showed different virulence to mice [5-7]. Katz reported that 9 of 15 H5N1 viruses isolated from humans in Hong Kong in 1997 were highly pathogenic (HP) to mice, whereas 5 of them exhibited a low pathogenic (LP) phenotype, replicating only in the respiratory tract without mortality. The remaining one strain showed an intermediate pathogenicity phenotype [7]. All 15 viruses shared a multi-basic amino acid (aa) motif at the cleavage site between HA1 and HA2 which was lethal for experimentally infected chickens [5,8,9]. One of the HP H5N1 viruses, A/Hong Kong/483/97, contained lysine at aa position 627 in the PB2 protein, whereas one of the LP H5N1 viruses, A/Hong Kong/486/97, contained glutamic acid at the same position, demonstrating that a single aa residue at position 627 was a key molecular determinant for virulence in mice [10]. However, when PB2 aa sequences were compared among the HP H5N1 viruses, only three of the 9 HP H5N1 viruses contained a lysine at PB2 aa residue 627 (627 K) [11,12]. Thus, PB2 aa 627 K alone did not correlate with lethality in mice, suggesting that other genetic variations were involved in virulence in mice but that this residue could not affect replicative efficiency in mice [13].

The high cleavability of the hemagglutinin glycoprotein (HA) was essential for lethal infection in birds, suggesting that the HA protein also plays an important role in the HP phenotype in humans. As HA mediates viral binding to host cell sialic acid-specific receptors and the subsequent fusion of the membrane of the endocytosed virus particles with the endosomal membrane leads to the release of vRNP into the cytoplasm, the cleavage site is associated with H5N1 pathogenicity [10]. Other studies suggested that 92 E of the NS1 protein is important for abrogating the antiviral effects of interferon and tumor necrosis factor alpha, and may be crucial to the pathogenicity in pigs [14]. A recent study demonstrated that aa residue 66 S of PB1-F2 affects the pathogenicity of an H5N1 virus in mice [15].

Since the SP/83/04 (SP83) strain isolated in Thailand in 2004 is LP to ferrets and mice *in vivo*, and the KAN/353/04 (KAN353) strain isolated in Thailand in the same year shows HP to ferrets [16], we used these viruses to characterize *in vitro *phenotypes associated with the pathogenicity in animals. We also used four H5N1 viruses isolated in Thailand in 2004 from humans. Although a reverse genetics system and animal experiments are needed to confirm the segments involved in the virulence, comparison of the *in vitro *phenotype described in this study may provide the basis for assessing the pathogenesis of influenza H5N1 viruses *in vivo*.

## Materials and methods

### Viruses and cells

Six H5N1 influenza viruses, SP83, KAN353, Thai/1623/04 (Thai1623), KK/494/04 (KK494), PCBR/2031/04 (PCBR2031), and SP/528/04 (SP528), isolated from humans in Thailand in 2004 were used in this study. The viruses were isolated with MDCK cells and grown once in 10-day-old embryonated chicken eggs. The allantoic fluid was used as the virus stock. MDCK cells were maintained in MEM supplemented with 10% newborn calf serum and antibiotics at 37°C in 5% CO_2_. All experiments were performed in a biosafety level 3 containment laboratory.

### Plaque assay

To measure the virus infectivity, we performed a plaque assay as described previously [17]. Briefly, MDCK cells were plated at 6 × 10^5^/well in 6-well microplates one day before the assay. The confluent cells were infected with serial 10-fold dilution of virus samples and incubated for 1 hr at 37°C with shaking every 15 min. The cells were washed with phosphate-buffered saline (PBS) and covered with 1% agarose in a 2 × MEM medium containing 5 μg/ml of TPCK-trypsin (Sigma, Missouri, USA). After incubation for 3 days at 37°C, the agarose was removed and the cells were fixed with 10% formaldehyde and then stained with 0.1% crystal violet to visualize the plaques. The infectivity titer was expressed by plaque-forming units (PFU).

### Real-time PCR

The viral RNA was extracted from the culture medium of MDCK cells or allantoic fluid by using a QIAamp viral RNA Mini kit (QIAGEN, Hilden, Germany). The RNA was reverse-transcribed to cDNA by using random primers (Invitrogen, Oslo, Norway). We used 5-μl portions of cDNA to amplify the M gene by real-time PCR using a forward primer A/M264R2 (5-ACAAAGCGTCTACGCTGCAG) and a reverse primer A/M30F (5-TTCTAACCGAGGTCGAAACG) as described previously http://www.nih.go.jp/niid/index-e.html (in Japanese). The amplification was performed by using SYBR Green (ABI, Warrington, UK) according to the method described previously [18] with slight modifications. The pretreatment of the reaction was carried out at 95°C for 10 min, then subjected to 40 cycles of amplification at 95°C for 15 sec and at 60°C for 1 min.

### Virus infection to embryonated eggs

Ten-day-old embryonated eggs were inoculated with the viruses and incubated at 37°C. The dead eggs were checked every 12 hours. PBS was used as the negative control. After 24 hours of infection, allantoic fluid was used for the virus titrations by plaque assay.

### Sequence analyses

Viral RNA extracted with a QIAamp viral RNA Mini kit was used in a one-step reverse transcription PCR (QIAGEN). The PCR products were cloned into the pGEM-T Easy Vector System (Promega, Madison, USA). The plasmid was extracted with the GenElute™ Plasmid Miniprep Kit (Sigma) and used for sequencing by the ABI BigDye terminator cycle-sequencing kit with an ABI 3100 Genetic Analyzer (Applied Biosystems, Foster City, CA, USA). Amino acid sequences were analyzed by BioEdit.

## Results

### H5N1 virus infection on embryonated eggs

The six avian influenza H5N1 viruses used in this study are shown in Table 1. Only one strain, SP528, was isolated from a patient who recovered; the other 5 strains were from patients who eventually died. Of these strains, SP83 and KAN353 have been examined previously for their pathogenicity *in vivo *using ferrets and mice, and the experiments showed that SP83 has low virulence, whereas KAN353 appears to be an HP virus [16]. To determine whether the pathogenicity of H5N1 viruses can be evaluated in an *in vitro *assay, we tested the availability of embryonated eggs. Each of 6 embryonated eggs was inoculated at 10 or 100 PFU/egg/100 μl of the viruses and incubated at 37°C. After 36 hours, all of the eggs infected with 100 PFU/egg died, indicating that 100 PFU/egg of the virus was not appropriate for the assay. All eggs inoculated with KAN353 and Thai1623 died even in the 10 PFU/egg infections, whereas two of six eggs inoculated with SP83 died after the 10 PFU/egg inoculation (Table 2). Four SP83-infected eggs did not die until 72 hours post infection (p.i.) (data not shown), suggesting that the sensitivity of embryonated eggs is depent upon the virus pathogenicity. Five of six eggs died when 10 PFU/egg of the KK494 and PCBR2031 strains were used, and three of six eggs died in the case of SP528.

**Table 1:** Viruses Used in This Study

Strain	Abbreviation	Age	Gender	Status	Reference
A/Thailand/SP83/2004	SP83	58	F	Dead	[16]
A/Thailand/Kan/353/2004	KAN353	6	M	Dead	[16]
A/Thailand/1623/2004	Thai1623	18	M	Dead	This study
A/Thailand/KK/494/2004	KK494	4	M	Dead	This study
A/Thailand/PCBR/2031/2004	PCBR2031	9	F	Dead	This study
A/Thailand/SP/528/2004	SP528	1	M	Recovered	This study

**Table 2:** Lethality of embryonated eggs after being infected with H5N1 36 hours p.i.

Inoculum	SP83	KAN353	Thai1623	KK494	PCBR2031	SP528
100PFU	6/6	6/6	6/6	6/6	6/6	6/6

10PFU	2/6	6/6	6/6	5/6	5/6	3/6

**Figure 1:**
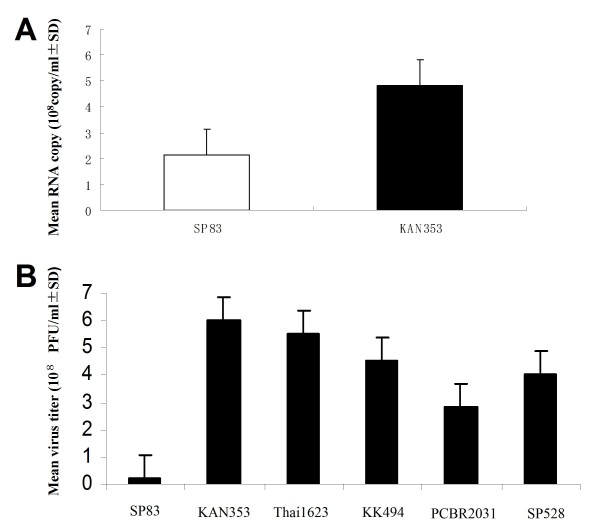
**H5N1 virus infection in embryonated eggs**. (A) Viral RNA copy numbers from embryonated eggs infected with 100 PFU/ml of SP83 and KAN353, 24 hours p.i. (B) Infectivity of allantoic fluid from embryonated eggs under the same experimental conditions.

To confirm the availability of embryonated eggs for the evaluation of pathogenesis, we compared the copy numbers of the viral genome by using the allantoic fluid collected 24 hours p.i. KAN353 and SP83 were used to inoculate the eggs at 100 PFU/egg. Real-time PCR indicated that the copy numbers of the RNA were much higher in the KAN353-infected eggs than in the SP83-infected eggs (Fig. 1A). When the infectivity of these viruses was measured by plaque assay, we found that the infectivity of SP83 was significantly lower compared with that of the other strains, especially KAN353 and Thai1623 (Fig. 1B). These results indicated that compared with SP83, KAN353 showed higher pathogenicity to embryonated eggs and replicated faster and more extensively in embryonated eggs.

### Virus replication in MDCK cells

**Figure 2:**
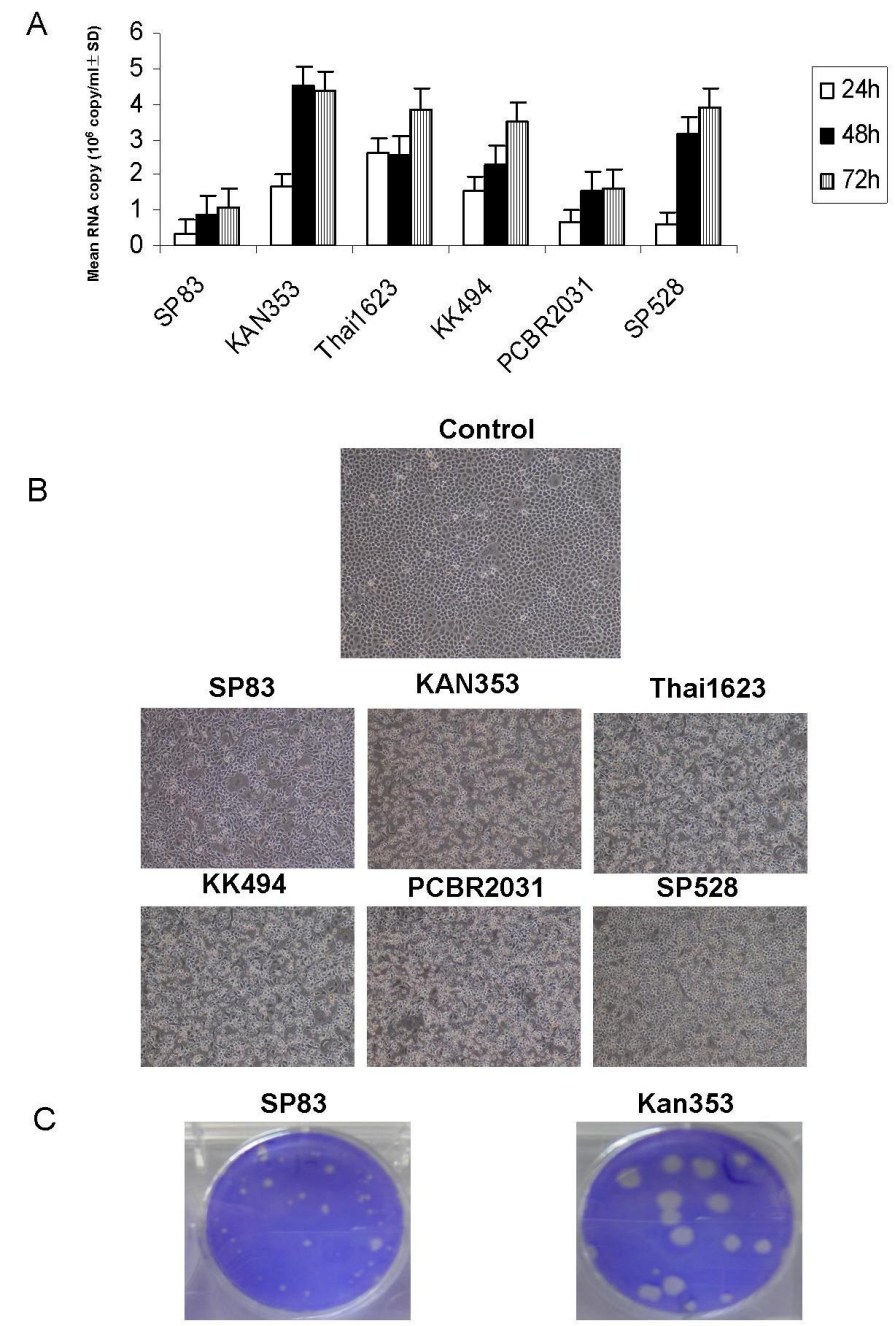
**Comparison of replication and cytopathic effect in MDCK cells**. (A) MDCK cells were infected with H5N1 viruses at MOI 1, and the copy numbers of viral RNA in the medium were measured by real-time PCR 24, 48, and 72 hours p.i. (B) Cytopathic effect on MDCK cells 24 hours p.i. Mock-infected cells were used as the negative control. (C) Plaques on MDCK cells infected with SP83 and KAN353 72 hours p.i.

To explore the availability of MDCK cells for evaluating the pathogenesis of influenza viruses, we infected the cells with H5N1 viruses at a multiplicity of infection (MOI) of 1 and incubated the cells at 37°C. After 24 hours, the copy numbers of the virus genome in the culture medium were measured by a real-time PCR. At the same time, the morphology of MDCK cells was monitored after infection. The copy number of the viral RNA in KAN353-infected cells was 1.7 × 10^6^, whereas in the SP83-infected cells it was 3.6 × 10^4^, indicating a 45-fold difference between high and low pathogenic strains. The RNA copy numbers 48 hours and 72 hours p.i. were 5 and 4 times higher in KAN353 than SP83, respectively (Fig. 2A). The copy numbers of the other four strains were 2.7 × 10^6^, 1.6 × 10^6^, 6.6 × 10^5^, and 6.0 × 10^5 ^in the Thai1623-, KK494-, PCBR2031-, and SP528-infected cells 24 hours p.i., respectively. The cytopathic effect of the virus of KAN353 was the most extensive, while SP83 showed the least cytopathic effect among the six strains in MDCK cells (Fig. 2B). MDCK cells infected with KAN353 showed large plaques with a mean size of 4.68 ± 0.15 mm, whereas SP83 produced significantly smaller plaques with an average size of 1.65 ± 0.15 mm (Student's t test; P < 0.001) (Fig. 2C). These results indicated that SP83 replicated more slowly and less extensively in MDCK cells and showed a weak cytopathic effect compared with KAN353.

### Amino acids changes related to pathogenicity

PB2 aa positions 627 and 701 are known to be related to virulence of the influenza virus in mammalian hosts, including mice and guinea pigs [10,13,19-23]. PB2 aa 627 E is related to low viral virulence, and this aa is observed in LP SP83 and SP528, whereas PB2 aa 627 in the other 4 strains including KAN353 is K, which is related to high virulence (Table 3). The PB2 aa 701 N is also known to be related to high virulence, and this aa is observed in SP528, whereas PB2 701 in the other 5 strains, including HP KAN353, is D, which is related to low virulence. A recent report indicated that transmission of an influenza virus in a mammalian host is increased by PB2 aa 627 K or a combination of PB2 aa 627E/701N [22], confirming that SP83 has low virulence, whereas KAN353 has high virulence, and suggesting that the other 4 strains, including SP528, have virulent phenotypes.

**Table 3:** Comparison of amino acids related to viral pathogenicity

		Pathogenicity							
Protein	Position	Low	High	SP83	KAN353	Thai1623	KK494	PCBR2031	SP528
PB2	627	E	K	E	K	K	K	K	E
	701	D	N	D	D	D	D	D	N
PB1-F2	66	N	S	N	N	N	N	N	N
NS1	92	D	E	E	E	E	E	E	E
Cterminus R-S-E-V R-S-E-V R-S-E-V R-S-E-V						R-S-E-V	R-S-E-V	R-S-E-V	R-S-E-V

PB1-F2 aa 66 N is known to be related to low pathogenicity, and an N-to-S substitution has been shown to contribute to increased virulence in mice [15]. However, none of the 6 viruses we tested had this substitution (Table 3). Similarly, NS1 aa 92 E and the C terminus ESEV are known to be related to the high pathogenicity of H5N1 [14,15,24,25]. However, these aa are conserved in all viruses. These results suggested that PB1-F2 aa 66, NS1 aa 92, and NS1 C terminus 4 aa residues alone may not determine the virulence. The amino acid differences in all segments of HP KAN353 and LP SP83 are shown in Table 4. We found 24 aa differences, and aa residues RERRRKR forming the HA cleavage site are conserved in KAN353 and SP83 (data not shown). No amino acid differences in PB1, NP, or M proteins were found between HP KAN353 and LP SP83. These results suggested that PB2 aa 627 may be related to virus phenotypes, as shown above; however, the effects of other amino acid differences on the phenotypes is unknown at present.

**Table 4:** Amino acid differences between SP83 and KAN353

Virus										Amino acid														
	**HA**					**PA**						**NA**							**PB2**					**NS**
					
	**314**	**370**	**380**	**417**	**529**	**142**	**163**	**200**	**216**	**278**	**628**	**9**	**17**	**44**	**74**	**102**	**180**	**361**	**192**	**355**	**443**	**627**	**710**	7

SP83	T	H	Y	S	I	K	P	A	D	Q	V	A	S	Q	V	T	S	I	D	R	R	E	I	S
KAN353	A	Y	C	N	V	R	L	T	N	R	M	T	T	H	I	I	N	T	E	K	K	K	T	P

## Discussion

Avian influenza H5N1 viruses continue to cause disease in poultry and humans in southeastern Asia http://www.who.int/csr/disease/avian_influenza/en/. The 2004 human H5N1 isolates were more virulent in the ferret model, causing severe systemic infection and rapid disease progression. The lethality was different between highly virulent 2004 H5N1 viruses and 1997 H5N1 viruses [16]. To better understand the potential of H5N1 viruses to cause disease in mammalians, we characterized 6 H5N1 strains isolated from humans in Thailand *in vitro. *Maines et al. experimented with SP83 and found that this strain has low virulence in ferrets compared to KAN353, which replicated to high titers in the respiratory tract of mice and ferrets, and was isolated from multiple organs, including the brain. In contrast, infection by SP83 occurred only in the respiratory tract in mice, and showed limited dissemination from the respiratory tract in ferrets [16]. In our study, we demonstrated that KAN353 showed HP to embryonated eggs and replicated quickly in Madin-Darby canine kidney (MDCK) cells, producing large plaques. In contrast, SP83 showed LP to embryonated eggs and replicated slowly in MDCK cells, producing small plaques.

As indicated by lethality in embryonated eggs, viral RNA copy in allantoic fluid, and virus titer measured by plaque assay, our results demonstrated that virulent KAN353 is highly pathogenic to embryonated eggs and replicated faster in embryonated eggs compared with nonvirulent SP83. In addition, virus growth measured by RNA copy numbers, cytopathic effect, and plaque size indicated that LP SP83 replicated slowly and less extensively in MDCK cells, and showed a weak cytopathic effect compared with HP KAN353. The relationship between plaque size and the pathogenicity of viruses is consistent with the results described previously, in which the A/Vietnam/1203/04 (H5N1) strain isolated from a human and grown in chicken embryos produced a heterogeneous virus population that formed two types of plaques in MDCK cells, differing in size and pathogenicity for ducks, ferrets, and mice. The viruses recovered from large plaques, like the wild-type, were highly pathogenic in ducks and mice, whereas those from small plaques were non-pathogenic in ducks and mice [26]. Plaque size may be an indicator for judging the pathogenicity of the H5N1 virus in *vivo*.

The aa position 627 of the PB2 protein was first recognized as a determinant of host range [19], and the 627 E-to-K substitution was identified as a molecular determinant of virulence in a pair of 1997 H5N1 viruses in inbred mice [10], although certain 1997 H5N1 viruses that lacked this substitution were also highly lethal for mice [7]. PB2 aa 627 is also suggested to increase the replicative efficiency in mouse cells, and the presence of K leads to more aggressive viral replication, overwhelming the host defense mechanisms and resulting in high mortality rates in mice [13]. A 627 K-to-E substitution has been shown to decrease the replication in primary mouse astrocytes and LA-4 mouse lung adenoma cells [13]. The residue 627 K may contribute to the large plaque size, high virulence to eggs, and fast replication of viruses. The SP528 used in this study has PB2 aa 627 E, which is associated with low virulence, and also has PB2 aa 701 N, which is associated with high pathogenicity [20,21]. However, these aa combinations are suggested to be associated with virulent phenotypes in a guinea pig model [22]. In fact, SP528 showed virulent phenotypes in our *vitro *assays. The mechanism of the effect of the PB2 aa residue 627 was described recently, in that the 627 K-to-E substitution resulted in a decreased association between PB2 and NP proteins, resulting in decreased genome transcription, replication, and virus production in primate cells [27]. We speculate that PB2 aa 627 may contribute to the phenotype of the H5N1 virus, but other segments of the virus gene may also contribute to the pathogenicity of the H5N1 virus.

Lycett et al. reported that combinations of aa residues at PB1-317/PB2-355, NS1-92/NS1-228, and HA-102/NS1-195 are related to the virulence of H5N1 viruses [28]; however, the viruses used in the present study did not show any relationship between these aa residues and virulent phenotype (Table 4). Mutations of PA-T515A and PB1-Y436H are also known to be involved in pathogenesis [26], but these aa residues alone were not associated with any virulent phenotype.

Animal experiments are needed to determine the pathogenicity of the four strains *in vivo*, and a reverse genetics system is needed to confirm which segment or which aa changes contribute to the phenotypes.

## Conclusions

The HP KAN353 strain showed fast replication and higher virulence in embryonated eggs compared to other strains, especially compared to the LP SP83 strain. HP KAN353 also showed strong cytopathogenicity compared to SP83 in Madin-Darby canine kidney cells. Interestingly, LP SP83 induced smaller plaques compared to other strains, especially HP KAN353. PB2 amino acid 627 E may contribute to low virulence, whereas either PB2 amino acid 627 K or the combination of 627E/701N seems to be associated with high virulence. The *in vitro *assays used in this study may provide the basis for assessing the pathogenesis of influenza H5N1 viruses *in vivo*.

## Competing interests

The authors declare that they have no competing interests.

## Authors' contributions

YGLI, MK, NT, KI, and PS designed this study. YGLI, MC, SW, YK and GRB carried out the experiments. YG.LI prepared the manuscript. All authors read and approved the final manuscript.
